# Prognostic value of preoperative modified Glasgow prognostic score in surgical non-small cell lung cancer: A meta-analysis

**DOI:** 10.3389/fsurg.2022.1094973

**Published:** 2023-01-09

**Authors:** Chenli Yang, Guangshu Ren, Qingqing Yang

**Affiliations:** ^1^ Department of Cardiothoracic Surgery, Gansu Provincial Hospital of TCM, Lanzhou, China; ^2^ Department of Thoracic Surgery, The 940th Hospital of Joint Logistics Support Force of Chinese People's Liberation Army, Lanzhou, China; ^3^ Department of Traditional Chinese Medicine, Gaolan Country People’s Hospital, Lanzhou, China

**Keywords:** modified Glasgow prognostic score, non-small cell lung cancer, surgical, prognosis, meta-analysis

## Abstract

**Background and purpose:**

The predictive role of modified Glasgow prognostic score (mGPS) for long-term survival in several types of cancers has been well manifested. We supposed that preoperative mGPS might also be associated with long-term survival of operated non-small cell lung cancer (NSCLC) patients. The aim of this meta-analysis was to identify the prognostic value of preoperative mGPS in surgical NSCLC patients.

**Methods:**

The PubMed, Web of Science, EMBASE and CNKI databases were searched for relevant studies up to November 7, 2022. The primary and secondary outcomes were overall survival (OS) and disease-free survival (DFS), respectively. The hazard ratios (HRs) and 95% confidence intervals (CIs) were combined.

**Results:**

A total of 3,803 patients from 11 studies were enrolled and analyzed. The combined results demonstrated elevated preoperative mGPS was significantly related to poorer OS (HR = 2.11, 95% CI: 1.83–2.44, *P* < 0.001) and DFS (HR = 1.70, 95% CI: 1.42–2.03, *P* < 0.001). Subgroup analysis for the OS further identified the predictive role of elevated preoperative mGPS for worse OS in NSCLC.

**Conclusion:**

Preoperative mGPS was significantly associated with prognosis in NSCLC and patients with elevated preoperative mGPS experienced poorer long-term survival.

## Introduction

Lung cancer is the most commonly diagnosed cancer and remains the leading cause of tumor-related death over the world (1, 2). Non-small cell lung cancer accounts for about 85% of all lung cancer cases (3). With the enhancement of public awareness of physical examination and popularization of low-dose spiral CT, the proportion and number of early-stage NSCLC patients have increased obviously in the past years and most NSCLC patients would receive surgical therapy (4). Up to now, the tumor-node-metastasis (TNM) stage system is still the most authoritative tool to assess the postoperative long-term survival and develop treatment and follow-up strategies for NSCLC patients. However, it is known that TNM stage is not sufficient to accurately predict the prognosis and a lot of factors play a role in predicting or affecting the survival of NSCLC patients. Thus, it is still necessary to explore and identify more valuable and reliable prognostic indicators contributing to the precise treatment of NSCLC patients.

Lots of evidence has proven that the systemic inflammation and nutritional status are significantly associated with the incidence, disease progression, therapy and prognosis of cancer patients (5–9). In the last 10 years, some indexes reflecting the systemic inflammation and nutritional status of the body based on peripheral blood indicators were described and reported to be related to the prognosis of NSCLC patients such as the C-reactive protein to albumin ratio (CAR) (10), albumin to globulin ratio (AGR) (11), and systemic inflammation response index (SII) (12). However, all these indexes are continuous variables and it is hard to define the normal or abnormal levels of individual patients. Another index, modified Glasgow prognostic score, is calculated based on C-reactive protein and albumin concentrations and categorized as 0, 1 and 2 (13). Patients with elevated C-reactive protein level (>0.5 mg/dl) and hypoalbuminemia (<3.5 g/dl) were assigned a mGPS of 2, while those with only elevated C-reactive protein level were assigned a mGPS of 1 and those with normal C-reactive level were assigned a mGPS of 0 (14). Thus, the mGPS is believed to serve as a more stable and reliable prognostic indicator in NSCLC compared with above mentioned indexes.

The prognostic value of mGPS has been widely verified in several types of cancers such as the gynecologic cancer, esophageal cancer, renal cell carcinoma and pancreatic cancer (15–18). As for NSCLC, several studies have revealed the relationship between mGPS and survival of NSCLC patients. However, most relevant studies focused on advanced NSCLC patients. Up to now, the prognostic role of preoperative mGPS in NSCLC patients receiving the surgical treatment remains unclear.

This meta-analysis aimed to further identify the predictive role of preoperative mGPS for long-term survival in NSCLC, which might contribute to the accurate assessment of postoperative prognosis of NSCLC patients.

## Materials and methods

This meta-analysis was performed according to the Preferred Reporting Items for Systematic Reviews and Meta-Analyses guidelines (2020) (19).

### Literature search

The PubMed, EMBASE, Web of Science and CNKI electronic databases were searched from inception to November 7, 2022. The following terms were used during the search: modified Glasgow prognostic score, mGPS, lung, pulmonary, tumor, cancer, carcinoma, neoplasm, survival, prognostic, prognosis, surgery, resection, surgical and operated. The detailed search strategy was as follows: (modified Glasgow prognostic score OR mGPS) AND (lung OR pulmonary) AND (tumor OR cancer OR carcinoma OR neoplasm) AND (survival OR prognostic OR prognosis) AND (surgery OR resection OR surgical OR operated). Besides, the references cited in included studies were also reviewed for availability.

### Inclusion and exclusion criteria

The inclusion criteria were as follows: (1) patients were diagnosed with primary NSCLC and received surgical therapy; (2) the levels of peripheral C-reactive proteins and albumins were detected before the surgery; (3) patients were divided into three groups according to the mGPS (mGPS 0, 1 or 2) and prognosis of patients were compared between groups; (4) the mGPS 0, 1 and 2 were defined as: elevated C-reactive protein level (>0.5 mg/dl) and hypoalbuminemia (<3.5 g/dl) were assigned a mGPS of 2, while those with only elevated C-reactive protein level were assigned a mGPS of 1 and those with normal C-reactive level were assigned a mGPS of 0; 5) the hazard ratios (HRs) and 95% confidence intervals (CIs) of overall survival (OS) or disease-free survival (DFS) were reported directly in articles.

The exclusion criteria were as follows: (1) letters, editorials, reviews, case reports or animal trials; (2) duplicated or overlapped data.

### Data extraction

The following information was extracted from each included studies: the name of first author, publication year, country, sample size, tumor-node-metastasis (TNM) stage, comparison of mGPS, endpoint, NOS score, HR and corresponding 95% CI.

### Methodological quality assessment

All included studies were retrospective. Thus, the Newcastle Ottawa scale (NOS) was applied to assess the quality of included studies and studies with a NOS score of 6 or higher were defined as high-quality studies (20).

The literature search, selection, data extraction and quality assessment were all performed by two authors independently and all disagreement was resolved by team discussion.

### Statistical analysis

The HR with 95% CI were combined to identify the association between preoperative mGPS and OS or (and) DFS of NSCLC patients. The heterogeneity among the included studies was evaluated by *I*^2^ statistics and Q tests. When significant heterogeneity was observed, presenting as *I*^2^ greater than 50% or *P* value less than 0.1, the random-effects model was applied; otherwise, the fixed-effects model was used (21). Subgroup analysis based on the country and comparison of mGPS were further conducted. Besides, sensitivity analysis was conducted to identify the source of heterogeneity and evaluate the stability of the pooled results. Furthermore, Begg's funnel plot and Egger's test were conducted to detect publication bias (22, 23). Significant publication bias was defined as *P* < 0.05. All statistical analysis in this meta-analysis was conducted by STATA 12.0 software.

## Results

### Literature search

**Figure 1:**
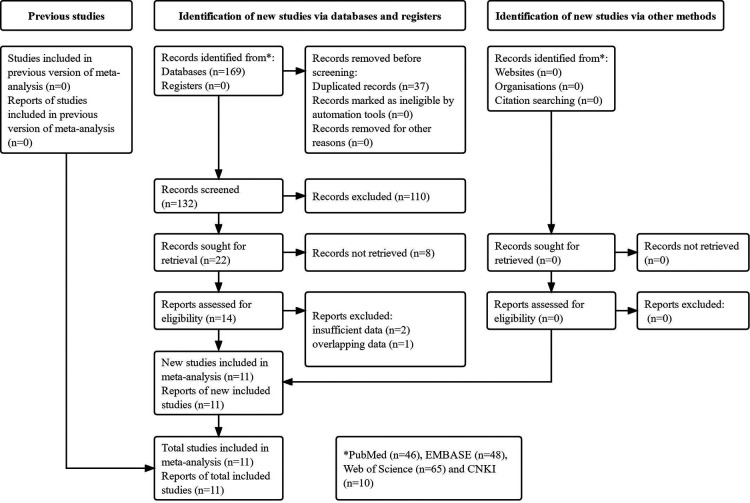
The flow diagram of this meta-analysis.

The detailed literature search process was presented in Figure 1. Initially, 169 records were identified from the electronic databases and 37 duplicated records were removed. Then 118 irrelevant or unavailable publications were excluded. The full texts of remaining 14 studies were reviewed. Finally, a total of 11 relevant studies were included in our meta-analysis (24–34).

### Basic characteristics of 11 included studies

A total of 3,803 patients from these 11 retrospective studies were enrolled with the sample size ranged from 63 to 1,243. Most of them were from Asian countries including the China and Japan. All included studies were defined as high-quality studies with a NOS score ≥6. Other characteristics were shown in Table 1.

**Table 1:** Basic characteristics of included studies.

Author	Year	Country	Sample size	TNM stage	Comparison of mGPS	Endpoint	NOS
Pinato (24)	2014	UK	220	I–IIIA	0/1/2	OS	6
Fan (25)	2016	China	1,243	I–IV	0/1/2	OS	7
Osugi (26)	2016	Japan	327	I–III	0–1/2	OS	7
Lv (27)	2017	China	266	I–III	0–1/2	DFS	6
Yamauchi (28)	2017	Germany	156	IIIA	0/2	DFS	6
Wang (29)	2020	China	139	I–IIIA	0/2	OS	7
Asakawa (30)	2021	Japan	286	I–IIA	0/1–2	OS, DFS	7
Matsubara (31)	2021	Japan	596	I–III	0/1–2	OS, DFS	6
Watanabe (32)	2021	Japan	387	I–III	0/1–2	DFS	6
Zhan (33)	2022	China	63	I–III	0/1–2	OS	7
Zhang (34)	2022	China	120	I–III	0/2	OS	7

TNM, tumor-node-metastasis; mGPS, modified Glasgow prognostic score; OS, overall survival; DFS, disease-free survival; NOS, Newcastle-Ottawa Scale.

### The association between preoperative mGPS and OS in NSCLC patients

**Figure 2:**
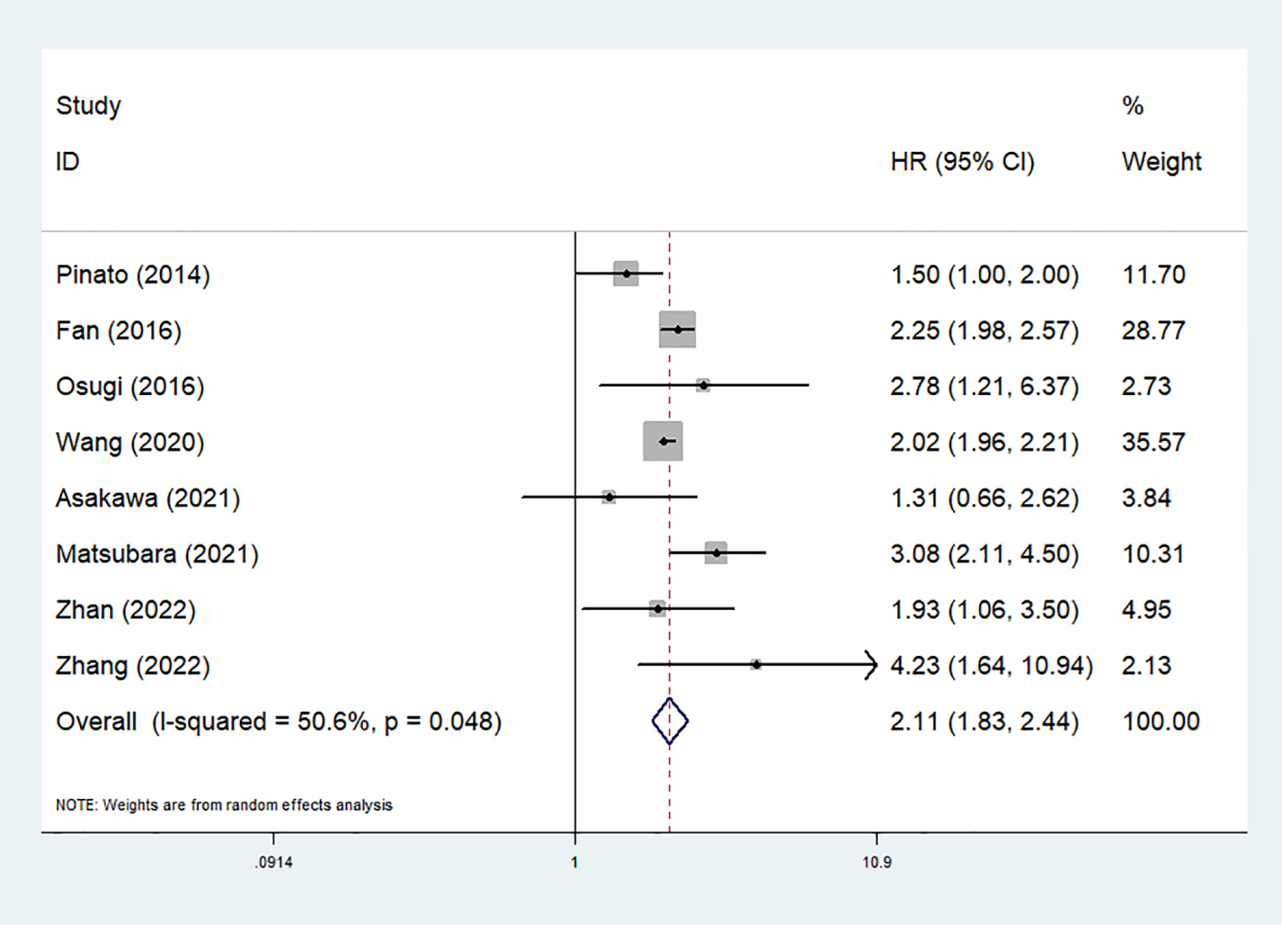
The association between preoperative modified Glasgow prognostic score and overall survival of surgical non-small cell lung cancer patients.

Eight studies explored the predictive role of preoperative mGPS for OS of NSCLC patients (24–26, 29–31, 33, 34). The pooled results demonstrated that an elevated preoperative mGPS was significantly related to poorer OS (HR = 2.11, 95% CI: 1.83–2.44, *P* < 0.001; *I*^2^ = 50.6%, *P* = 0.048) (Figure 2).

Then subgroup analysis based on the country and comparison of mGPS were further conducted. The results indicated that both non-Chinese (HR = 2.02, 95% CI: 1.28–3.20, *P* = 0.003) or Chinese (HR = 1.81, 95% CI: 1.13–2.88, *P* = 0.013) patients with an elevated preoperative mGPS had a worse OS. Similarly, the subgroup analysis stratified by the comparison of mGPS also further investigated the predictive role of preoperative mGPS for OS (0 vs. 1 vs. 2: HR = 1.90, 95% CI: 1.28–2.82, *P* = 0.001; 0–1 vs. 2: HR = 2.78, 95% CI: 1.21–6.37, *P* = 0.016; 0 vs. 1–2: HR = 2.23, 95% CI: 1.68–2.94, *P* < 0.001) (Table 2).

**Table 2:** Results of meta-analysis.

	No. of studies	Hazard ratio	95% confidence interval	*P* value	*I*^2^ (%)	*P* value
Overall survival	8 (24–26, 29–31, 33, 34)	2.11	1.83–2.44	<0.001	50.6	0.048
Country						
Non-China	4 (24, 26, 30, 31)	2.02	1.28–3.20	0.003	69.0	0.021
China	4 (25, 29, 33, 34)	1.81	1.13–2.88	0.013	79.4	0.002
Comparison of mGPS						
0 vs. 1 vs. 2	2 (24, 25)	1.90	1.28–2.82	0.001	78.4	0.031
0–1 vs. 2	1 (26)	2.78	1.21–6.37	0.016	-	-
0 vs. 1–2	2 (29–31, 33, 34)	2.23	1.68–2.94	<0.001	53.2	0.074
Disease-free survival	5 (27, 28, 30–32)	1.70	1.42–2.03	<0.001	48.3	0.102

mGPS, modified Glasgow prognostic score.

### The association between preoperative mGPS and DFS in NSCLC patients

**Figure 3:**
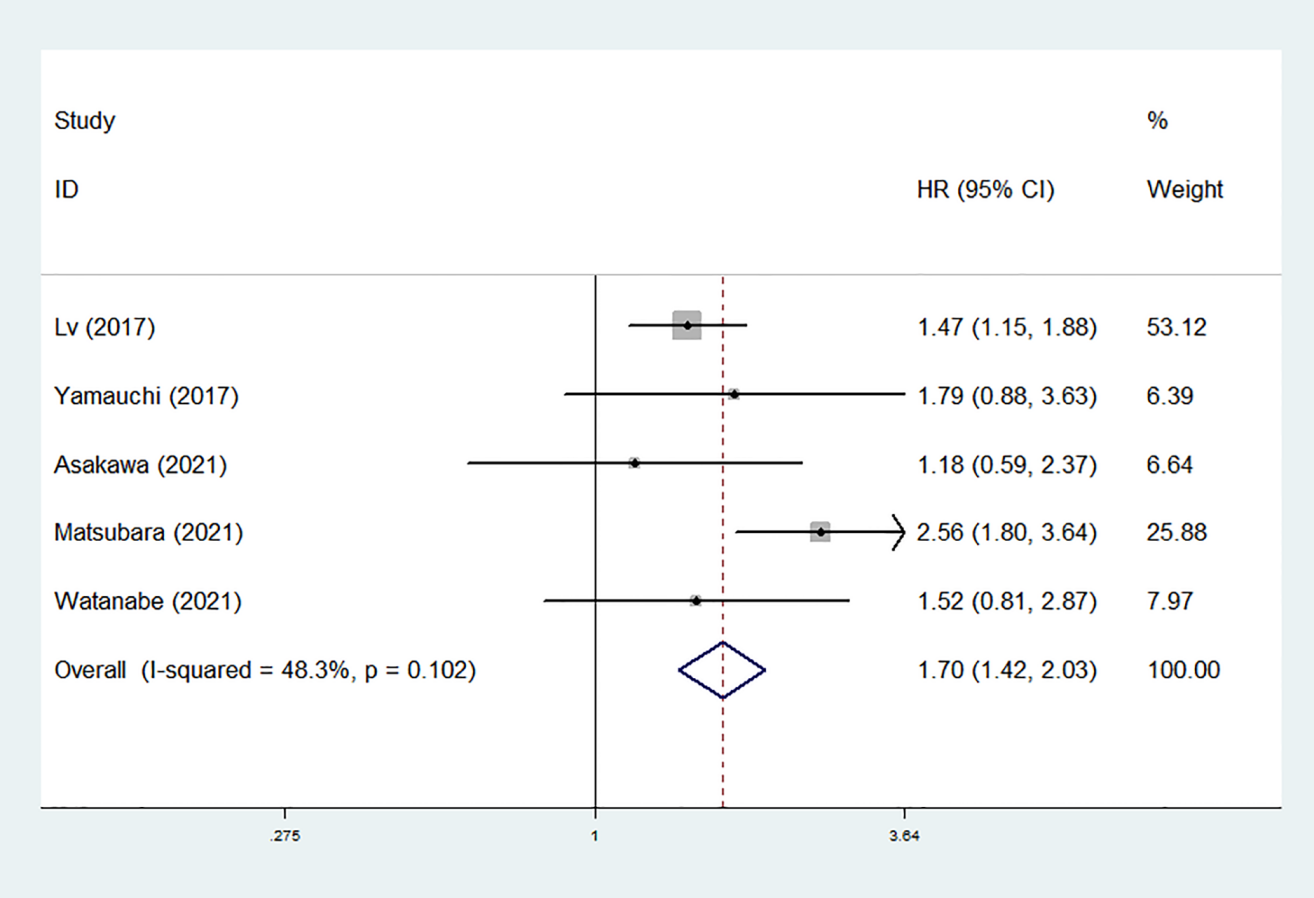
The association between preoperative modified Glasgow prognostic score and disease-free survival of surgical non-small cell lung cancer patients.

Five studies explored the relationship between preoperative mGPS and DFS in NSCLC (27, 28, 30–32). The pooled results manifested that elevated mGPS was significantly associated with poor DFS (HR = 1.70, 95% CI: 1.42–2.03, *P* < 0.001; *I*^2^ = 48.3%, *P* = 0.102) (Figure 3).

### Sensitivity analysis and publication bias

**Figure 4:**
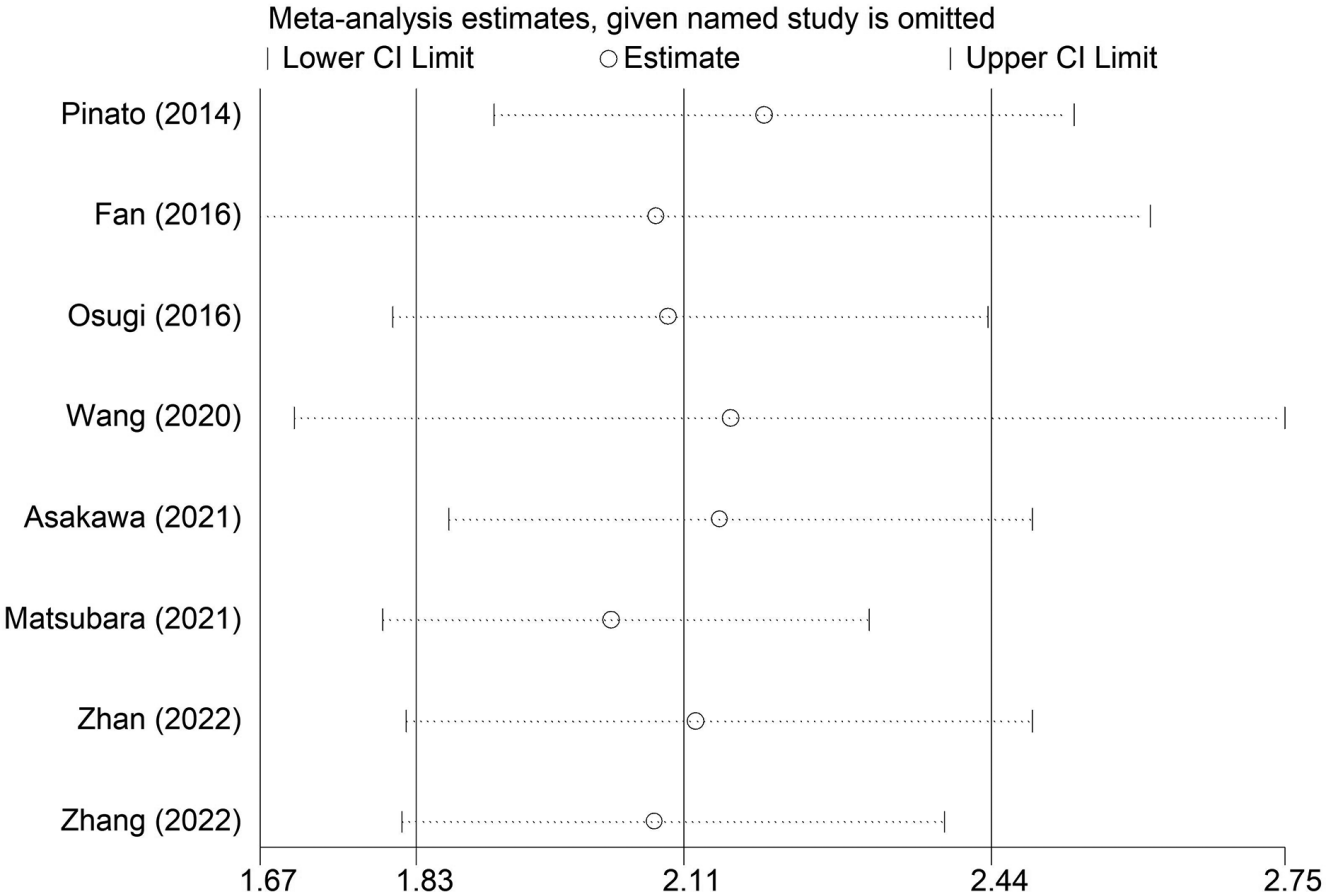
Sensitivity analysis for the association between preoperative modified Glasgow prognostic score and overall survival of surgical non-small cell lung cancer patients.

**Figure 5:**
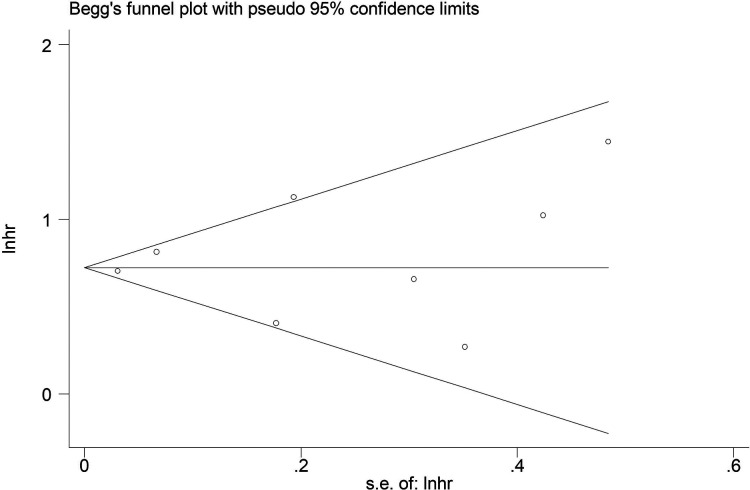
Begg's funnel plot.

The sensitivity analysis was conducted by excluding each included studies at each time, which indicated that the results of this meta-analysis were stable and reliable and none of included studies had a significant impact on the overall results (Figure 4). According to the Begg's funnel plot (Figure 5) and Egger's test (*P* = 0.621), no obvious publication bias was detected.

## Discussion

The current meta-analysis identified preoperative mGPS as a prognostic factor in NSCLC and patients with an elevated preoperative mGPS experienced obviously poorer OS and DFS compared with patients with a normal mGPS. Subgroup analysis based on the country and comparison of mGPS further manifested the prognostic value of preoperative mGPS in NSCLC patients. Thus, preoperative mGPS could serve as a novel and reliable predictor for long-term survival in surgical NSCLC patients. Patients with elevated mGPS might be followed up closely and receive more active treatment, but this should be further explored in future relevant studies. Due to the limitations existed in included studies and this meta-analysis, more prospective high-quality relevant studies are still needed to further verify the prognostic role of mGPS in NSCLC patients.

The prognosis value of mGPS has been well identified in several types of cancers. Wu et al. included 4,629 patients from 25 relevant studies and showed that elevated mGPS was related to poor OS in pancreatic cancer patients (HR = 1.92, 95% CI: 1.60–2.30, *P* < 0.002) (18). Besides, subgroup analysis based on the study design, region, disease status, treatment, cancer type, and study center indicated similar results (18). Wang et al. analyzed 3,415 cases from ten studies and indicated the significant association between elevated mGPS and poor OS (HR = 1.66, 95% CI: 1.14–2.41, *P* = 0.008) of esophageal cancer patients (13). Furthermore, another meta-analysis by Chen et al. revealed that mGPS was an independent risk marker of poor prognosis in hepatocellular carcinoma patients (HR = 2.21, 95% CI: 1.73–2.82) after reviewing 2,047 patients of seven relevant studies (35). Besides, Hu et al. enrolled 2,391 renal cell carcinoma patients for quantitative synthesis and demonstrated that higher mGPS was obviously related to poor OS (HR = 4.31, 95% CI: 2.78–6.68, *P* < 0.001), cancer-specific survival (HR = 5.88, 95% CI: 3.93–8.78, *P* < 0.001) recurrence -free survival (HR = 3.15, 95% CI: 2.07–4.79, *P* < 0.001) and progression-free survival (PFS) (HR = 1.91, 95% CI: 1.27–2.89, *P* = 0.002) (36).

Actually, two meta-analyses investigated the predictive role of mGPS for prognosis of lung cancer patients. Zhang et al. included 1,164 patients and manifested that elevated mGPS was related to poorer OS (HR = 4.61, 95% CI: 1.25–16.99, *P* = 0.022) and PFS (HR = 2.61, 95% CI: 1.28–5.34, *P* = 0.008) in advanced lung cancer patients receiving immune checkpoint inhibitor efficacy (37). The other meta-analysis by Jin et al. showed that elevated mGPS predicted OS (HR = 1.77, 95% CI: 1.35–2.31, *P* < 0.05) in all lung cancer patients, but a non-significant correlation between mGPS and OS in patients undergoing surgery was observed (HR = 2.48, 95% CI: 0.90–6.85, *P* = 0.079) (38). However, only three studies were analyzed in the subgroup analysis. The current meta-analysis included 11 studies and focused on NSCLC patients who received the surgery. This is the first to verify the prognostic value of preoperative mGPS in surgical NSCLC patients.

There are several limitations in our meta-analysis. First, all included studies are retrospective and the overall sample size was relatively small, which might cause some bias. Second, most of enrolled patients are from China or Japan, which might limit the generalizability of our conclusions. Third, we are unable to conduct subgroup analysis based on other important parameters such as the TNM stage, age, gender and postoperative adjuvant therapy due to the lack of original data. Four, only the association between preoperative mGPS and survival was investigated, the prognostic value of the change of mGPS during anti-tumor treatment should be explored in future studies.

## Conclusion

Preoperative mGPS was significantly associated with prognosis in NSCLC and patients with an elevated preoperative mGPS experienced worse long-term survival. However, more prospective high-quality relevant studies are still needed to further verify the prognostic role of mGPS in NSCLC patients.
